# Building the Language of Eternity

**Published:** 1880-07

**Authors:** Ausburn Towner


### BUILDING THE LANGUAGE OF ETERNITY.

AUSBURN TOWNER.

The language that we speak can have no more fitting comparison than to be likened to a vigorous, ever blossoming tree. It may have an humble origin, but its roots are firmly imbedded in the earth and the changes of centuries have only the more immovably fixed them. The Anglo-Saxon tongue forms the roots and trunk, the life and fibre, and whatever may be engrafted upon it, is moulded into a likeness to the parent tree and assumes at once, all of its characteristics and peculiarities. Since the time of William the Conqueror in England, more than eight centuries ago, our language, then a mere, uncertain twig, has been growing and changing, not in its general characteristics, or so that it can not be recognized, but like an oak or a pine, that is an oak or a pine when it is a mere shrub as well as when its girth becomes gigantic or its height towers over all the trees of the forest. The growth and change has never ceased. It is, perhaps, imperceptible when regarded in brief periods or seasons, but noticeable when observed in cycles measured by even a time less than that of a natural life. There are few readers of the BISTOURY who could not, with a slight effort of their recollection, bring to mind words that have changed in their signification or orthography in their own time, and a multitude of terms that have been incorporated into the language and become a part of it, since their school days. It would be trite to give examples of either the one or the other of these and would imply an uncomplimentary lack of perception on the part of the reader.[1]

These changes and improvements indicate a language that is feverishly alive and active, as much as the growth of a tree indicates a living and constantly enlarging creation.

I have often imagined how startled Shakespeare, Chaucer, Spencer or any of those men of genius, who laid the foundations of our language would be, if they could "revisit the glimpses of the moon" and hear their language spoken as it is spoken to-day. Whether this or the changes in the material appearance of the localities with which they were familiar, would the more disturb them, it would be hard to say. They certainly would have to struggle with 'vaccination,' 'telegraph,' 'telephone,' 'railroad,' 'locomotive,' 'photograph' and a thousand other words as much as does a nineteenth century American with such a stanzas as this:

Yt felle abought the Lamasse tyde
When husbonds wynn ther haye
That daughte Dowglass bowynd hym to ryde
In Ynglande to take a praye. 2

Yet the words and the verse are both good English.

The changes in words and their significations in the past two hundred and sixty-nine years, can be shown very clearly by an examination of the Bible, the version now commonly in use being in the English of that number of years ago. A careful examination will give a long

1. The flexibility of our language is not the least of its numerous strong qualities. The coinage of a new word is a matter of almost daily occurrence, and it is seen in the commonest affairs of life as well as in the most scientific or abstract investigations. Let a man invent or discover a new application of electricity or desire a striking name for a soap or an oil, he has the whole range of two languages that are fixed, because they are dead, before him to select from. He makes his choice from what might be called the dead echoes that are lying about the Ægean islands or the hills of Rome, breathes into it the breath of life and in the latter case at least, the term becomes so much a part of our every day speech that it appears on all the blank walls and high rocks that are accessible to a man with a brush and a pot of black paint. The genius of no other language is competent for such growth and expansion. Even the German which is the nearest allied to our tongue in some respects, naturalizes only with much difficulty terms that have their roots in another soil. It won't go to the Greek as we do, to name a telescope, but calls that instrument a Farseer, and declines to count in Latin, denominating its millionth, der zehnhunderttausendste, or the ten hundred thousandth. We could hardly get along and could reasonably expect but little of our language if we called so common a tradeswoman as a milliner by the extended term die Modewaarenhandlerinn,

There are other and perhaps less creditable methods of introducing new words into the English language. An editor or a proof-reader who may want to save a line, attract attention or create a sensation, will suddenly transform a good honest substantive into a rather shaky verb. He may be too lazy or not have enough room to say that some unfortunate person committed suicide and so he writes suicided instead; or in giving an account of a thief breaking into a house say he burgled it; or in speaking of a marriage ceremony conclude that the clergyman did not marry the couple and write that he marrified them. The savant, the student or the lexicographer first sees the word with horror and refuses to acknowledge it, but the kindly language with a motherly interest in a child that is lame or sickly or blind, takes to it gently and the next edition of the unabridged must contain it even if it is stamped as vulgar, or the publication is deemed behind the age.

2—This is a verse from a famous old English ballad called "The Battle of Otterbourne," that describes a hot conflict in which Shakespeare's "Hotspur" took part in 1387. In a modern way it would go something like this:

It fell about the Lammastide
When farmers mow their hay
That mighty Douglass prepared to ride
In England to take prey.

Lammas tide was one of four noted pagan feasts of ancient Britain that fell on August 1. The other three were February 1, May 1, and November 1.

list, much greater than one would suppose at a casual glance, for most of us are accustomed to read the Bible or to hear it read so frequently, that it seems to be written in precisely the same tongue in which we speak. Some of these words, have, in some unaccountable manner, come to mean precisely the opposite that they did when they were written, and others have so grown out of the ordinary use that they are seldom, if ever, seen anywhere save in the Bible. "Cunning" found many times between Genesis and Revelations, when used now-a-days, hardly means an aptness, an ingenuity, or a skillfulness such as is meant by Saul's servant when he says : "Behold I have seen a son of Jesse that is *cunning* in playing."[3] As we use the term, David would not have felt complimented and it is by no means in harmony with the words following immediately after those quoted. The verb, "prevent" meaning literally as it does only to go before, or to precede in most of the passages where it is used in the Bible, expresses now something a good deal beyond that mild action. "Glistering"[4] a good strong epithet, meaning the same as glistening, only intensified, has passed entirely out of use, as has also the verb "hale,"[5] to drag or draw violently or forcibly; and also "trow,"[6] to believe ; "doth" a form of the verb do ; "meet"[7] signifying fit ; "witty"[8] in the sense of ingenious and "wist"[9] one of the most attractive and poetical of verbs, meaning to know. "Poll,"[10] for head, is of frequent use in the Bible, but is now lost except in the legal term poll-tax, or, in the expression polled as applied to enumerating persons or questioning a jury. How it was ever twisted about to signify a place for holding an election, the acutest etymologist can hardly discover. "Polled" in the Bible, also means to have one's hair cut.[11]

Numerous other words could be added to these, given only as examples, if I was writing a book rather than a brief essay and there are some so strong and expressive that it seems a pity to see them passing out of general use.[12]

Many of these words are becoming obsolete because the occupation that gave rise to them has passed away. Distaff, for instance, is so seldom used, even in poetry, that its meaning is almost lost. It draws a picture instantly to the mind of one acquainted with it, of the most primitive times, the days of legend, tradition and historical obscurity. It might be observed here, that the words we may have lost in this wise, have been more than recompensed to us, by the introduction of a multitude of other terms rendered necessary by the rapidly accumulating inventions and discoveries of the present generation.

A number of other words have disappeared as we have become farther and farther removed by time from the original source of our language and the german manner of providing new words by compounding common and old ones, like furthermore, forasmuch, likeminded, loving-kindness, inasmuch and thankworthy. Also by the formation of the plural by adding n instead of s as hosen or housen as we still call more than one ox, oxen.[13]

Still more notable examples of these changes, inasmuch as the time is much farther extended, can be found in Shakespeare or Chaucer, by any one taking the trouble to look for them.[14]

The one tendency observable in all of these changes is, to simplify the language in every way; to so construct it that general rules may be made regarding it, with fewer and fewer exceptions to them. This, by no settled purpose, as the work of a committee who had set about framing a perfect tongue but by the combined sense of the many millions speaking and writing it. The old Greek language, the every day speech of the countrymen and contemporaries of Homer and Sophocles, was the most perfect tongue that the world, so far as we know

3—I Sam. XVI-18.

4—"The *glistering* sword cometh out of his gall"—Job XX-25.

5—"Lest thine adversary *hale* thee to the judge"—Luke XII-58.

6—"Doth he thank that servant because he did the things that were commanded him ? I *trow* not,"—Luke XVII-9.

7—"It is not meet so to do"—Ex. VIII-26.

8—"——and find out knowledge of witty inventions."—Prov. VIII-12.

9—"Wist ye not that I must be about my father's business ?" Luke II-49.

10—The nearest modern approach to its use, is in the poem "John Anderson my Joe" where his "frosty pow" is spoken of being probably Scotch for the term.

11—It seems from 2 Sam. XIV-26 that Absalom had his hair cut once a year. "And when he *polled* his hair, he weighed the hair——." He should have gone to his barbers just before he took his memorable ride on the mule.

12—A few of these are as follows : Countervail, damsel, delightsome, noisome, embolden, nethermost, wrought, thankworthy, molten, doting, fleshly, seemly, firstling, lusty, peradventure, froward, gainsay, fatling, dureth.

13—What's the objection to saying oxes, the same as axes or houses ?

14—As for instance, knave and villain. Neither of these terms originally carried any meaning beyond servitude and indicated nothing disgraceful. Remorseful used to mean pitiful; quote, to observe ; let, to hinder ; clerkly, scholarly ; wood, mad ; quick, lively ; tall, brave ; fantastical, supernatural ; bay, bark ; wot, to know and weeds, clothing. Then where have such words as these gone: gramercye, I thank you ; quhen, when ; quhat, what ; dinge, knock ; dole, sorrow ; banning, cursing ; dint, a blow ; busk, dress; and a multitude of others ?

it, was ever acquainted with. It is not at all doubtful that its formation and perfection was the work of many more centuries than the English language has been spoken, nor is it any more doubtful that when our language is as old as the Greek was when it was eclipsed by the Latin, it will be much nearer absolute perfection and better adapted to all of the purposes for which mankind uses speech.

In the orthographical construction of the words of our language, the changes have been quite as remarkable, if not more so, than in their signification. Take the verse printed above as an example. It might please the type setter, paper makers and ink manufacturers to spell "about" a-b-o-w-g-h-t, or "bound," b-o-w-y-n-d, or "prey" p-r-a-y-e, but it would satisfy no one else.[15] There has been a constant lopping off and expunging of letters. The old "felle" dropped the final e as useless and became fell. Man used to be spelled m-a-n-e, and dwell, d-w-e-l-l-e. Hundreds of words had that final e that was no more necessary than a tail to a man. You can hardly mention a substantive now ending with a consonant that was not so laden. But they all came off. The u came out of words ending in o-u-r, andthe k from words ending in c-k, the latter, however, not without a severe struggle. Some old-fashioned persons even now, insist on spelling "vigor" v-i-g-o-u-r, "color" c-o-l-o-u-r, "honor" h-o-n-o-u-r, "error" e-r-r-o-u-r, "traffic" t-r-a-f-f-i-c-k, and "music" m-u-s-i-c-k. The effort to drop the final letters in such words as programme, quartette, parquette and a number of others, that is being made now seems to be attended with similar difficulties.

In grammatical construction also, there are material changes. The number of irregular verbs is constantly decreasing, the participles and tenses taking a constant termination to the root. We say now help, helped, helping, where it used to be holpen, and climbed where even cultured persons used to say clumb or clomb. If we proceed, that most aggravating of irregular verbs "to be," must fall in with the others and instead of being a guerilla among words, will become a well drilled private in the ranks. I know of no good reason why a child's natural expression, "I be" is not just as good as "I am." Its offence is, after all, only against an entirely arbitrary rule of grammar.

While I sympathise heartily with the effort of those persons who are endeavoring to improve the language by making the spelling conform more nearly to the sound of the letters, I can not help but think that they will have their labor for their pains. A man or a class of men who undertake to make a wholesale change in a language at one stroke, will soon discover that they have rather a gigantic job on hand.[16]

Changes come gradually and by piece meal, and even if they are improvements will find obstinate ones who will cling to the ways of their fathers as long as they live, in the face of the whole world. If a publication of high standing and unquestionable scholarship sets the example of a certain way of spelling or a certain form of a verb and sticks to it in spite of ridicule and chaff, it will be a very short time before the change, if it be one founded in good sense will be an accepted one and be adopted into the language. No amount of legislative or congressional enactments can bring about such a result. The language will continue to change, improve and be enlarged, but it will be by the slow process of nature as heretofore and not by forcing

15—In the next verse of the ballad, without is spelled w-i-t-h-o-w-g-h-t-e-n, strife, s-t-r-y-f-f-e, and earl, y-e-r-l-l-e.

16—I wanted to say that, "they had bitten off more than they could chew," it does so cover the whole ground.

